# Case of Caries of the Os Coronary and Os Pedis

**Published:** 1880-10

**Authors:** William Soula


### ABSTRACT AND SUMMARY.

### CASE DEPARTMENT.

### A CASE OF CARIES OF THE OS CORONARY AND OS PEDIS, WITH FRACTURE OF THE SEVENTH RIB.

By William Soula.

A sorrel horse, æt 9; weight about eleven hundred pounds. The disease in this case was supposed to have originated from the prick of a nail in the foot. The history of the case, up to six days prior to death, was incomplete; further than this, that the horse had been disabled for some time. Six days before death, suppuration at the coronary band was diagnosticated. An incision was made at the anterior portion of the coronary band, and one half ounce of pus evacuated. After this warm fomentations were applied to the foot daily. The wound was injected twice a day with a preparation of Calendula, twenty minims to the ounce of water. The disease of the foot improved rapidly under this treatment, and at the end of four days the horse was able to bear the weight of the body on that foot, which it had not done before in some time. Two days after this severe constitutional symptoms developed. The pulse became rapid, full, and strong. The temperature rose to 105° F., and the respirations to forty per minute. The urine became thick, very dark in color, and was voided nearly every hour. Tr. of aconite was administered every hour to reduce the temperature. The following morning at 4 o'clock A.M., the horse died.

A necropsy was held on the horse the same morning, in the presence of Dr. Wm. H. Porter.

The foot was carefully examined, and all the disease that could be found about the foot was a fibrous thickening at the coronary band; there was a slight amount of caries around the margin of the lower articular surface of coronary bone, with caries of the opposing articular surface of the os pedis.

The lesion found in the foot did not seem serious enough to account for the sudden constitutional symptoms, and a further search was made.

*Thoracic Cavity.*—The heart was normal. A fracture of the seventh rib, on the left side, at its middle portion, was discovered. The broken ends of the rib were denuded of periosteum for an inch or more back of the fracture.

No attempt at repair was visible The broken ends of the rib were pretty firmly bound to the under-lying consolidated lung, by a thick layer of fibro-plastic, and fibrinous material. The lung in the immediate vicinity of the fracture, and for several inches around, was consolidated, and in a state of advanced cheesy degeneration. The non-consolidated portion of the left lung was oedematous The right lung was in a state hypostatic congestion.

The liver, spleen, and intestines were normal.

The capsule of the kidney was not adherent. Both kidneys were deeply congested, and a slight amount of pus was found in each pelvis. Both kidneys were in an advanced state of parenchymatous degeneration.

There were no metastatic abscesses, unless the one at the coronary band may be considered as such.

The case is quite an interesting one, and would be more so could it be positively determined, which was primary, the caries of the bones of the foot or the fracture. If the fracture of the rib, with the accompaning pleuro-pneumonia and cheesy degeneration of the consolidated lung was primary; it might be reasonable to infer that the foot trouble was of metastatic origin. The sudden developement of constitutional symptoms are explained, by an increase of the circumscibed pneumonia, and the congestion of the kidneys. The question arises in reviewing this case, Are horses likely to fracture a rib, or ribs, while being treated in slings?

